# Liraglutide effect in reducing HbA1c and weight in Arab population with type2 diabetes, a prospective observational trial

**DOI:** 10.1186/s40200-015-0178-6

**Published:** 2015-05-30

**Authors:** Alaaeldin M. K. Bashier, Azza Abdulaziz Khalifa Bin Hussain, Elamin Ibrahim Elamin Abdelgadir, Ahmed Tarig Eltinay, Puja Thadani, Mohamed Elhassan Abdalla, Salah Abusnana, Fatheya F. AlAwadi

**Affiliations:** Endocrine Division, Dubai Hospital, Dubai, UAE; Internal Medicine, Dubai Hospital, Dubai, UAE; Sharjah University, Sharjah, UAE; RCDR Centre, Ajman, UAE

**Keywords:** Liraglutide, Arab population, HbA1c, Weight, Blood pressure, Type2 diabetes mellitus

## Abstract

**Background:**

The pathophysiology of type2 diabetes differs between different ethnic groups. Asians develop type2 diabetes at younger age, lower body mass index, and in relatively short time. Not only that, some ethnicities have different responses and dosing regimens to different classes of anti-diabetic agents. Data from Japanese population showed that the optimal doses of liraglutide used are smaller than other population and that weight loss is not as effective as seen in Caucasians.

**Methods:**

We aimed to assess liraglutide efficacy in reducing weight and HbA1c in Arab population when used as add on to other anti-diabetic agents. We prospectively followed patients who were recruited to treatment with liraglutide for a 6 months period; at the start of the study we checked patients’ demographics, weight, blood pressure, fasting blood glucose, HbA1c, lipid panel, LFTs and creatinine. Patients were checked at 3 months and at the end of the study at 6 months.

**Results:**

There was a significant reduction in weight at 3 and 6 months from a mean weight of 96.01 ± 19.2 kg to (94.8 ± 20 kg with (*P* < 0.001)) and 94.5 ± 19 kg with (*p* < 0.001) respectively. Mean HbA1c at baseline was 8.3 ± 1.7 % dropped to 7.7 ± 1.4 % (*p* < 0.001) at 3 months, and 7.6 + 1.6 % (*p* < 0.001) at 6 months.

**Conclusions:**

Liraglutide is effective in reducing weight, HbA1c as well as other metabolic parameters in Arab population with type2 diabetes.

**Trial registration:**

The trial is approved and registered with the Institutional Ethical Committee Board (Dubai Health Authority Medical Research Committee) under registration Number (MRC-08/2013_03).

## Introduction

In healthy individuals the delicate balance between beta and alpha cells maintains the normal glucose homeostasis; elevated blood glucose results in inhibition of alpha cell production of glucagon and stimulation of beta cell production of insulin. This balance is disturbed in patients with diabetes, resulting in loss of first phase insulin response and increased glucagon levels [1, 2].

GLP-1 is a hormone produced in the gastrointestinal tract in response to oral meal; it stimulates insulin secretion, and inhibits glucagon production both in a glucose dependent manner. It also delays gastric emptying, and promotes satiety and hence results in improved post-prandial glycaemic control [3]. Liraglutide is a GLP-1 analogue that has 97 % structural homology to native GLP-1 and is used in the treatment of type2 diabetes as it has proved effectiveness in all LEAD trials (monotherapy or combination therapy) in reducing HbA1c and weight in all patients regardless of their ethnicity, weight and sex [3].

The pathophysiology of type2 diabetes differs between ethnic groups leading to a diverse clinical presentation. Asians develop diabetes in a relatively short time, in younger population, and with lower body mass index compared to other ethnic groups [4]. Many studies have evaluated the pathophysiologic defects in Asians; some have suggested that impaired insulin secretion as the main defect based on the fact that the insulin secreting capacity in Japanese is only 50 % of that observed in Caucasians [5, 6]. Other studies found a more pronounced incretin defect in Asians than in Caucasians [5]. The DECODE-DECODA study showed that the pathophysiologic defect in Caucasians is mainly a higher insulin resistance [6].

These differences in pathophysiology might explain different responses to some anti-diabetic medications. In one study that assessed glucagon response to liraglutide injection showed that it did not cause significant reductions in fasting or post prandial glucagon levels in Japanese type2 diabetics. This finding was attributed to the alpha cell characteristic of being affected by heterogeneity of the pathogenesis of diabetes, although the smaller dose used for Japanese population could be a contributing factor [3].

There were very little in literature to evaluate the effect of liraglutide in different ethnic groups; studies in Japanese population showed that liraglutide as monotherapy results in −2.34 kg weight reduction [7], but are weight neutral when used in combination with other oral anti-diabetic agents [4, 8]. Furthermore smaller doses of liraglutide (0.1–0.3 mg qd) did not result in improvement in HOMA-IR and HOMA-B, while higher doses (0.6 0.9 mg qd) resulted in (−0.32 and −0.53) reduction in HOMA-IR and increased HOMA-B (22 and 21.05 respectively) [7]. Another two studies have shown that liraglutide has a superior effect in reducing HbA1c in Asian population than in Caucasians.

The prevalence of diabetes and obesity is very high in the Arab countries, and according to last IDF (International Diabetes Federation) data the prevalence in United Arab Emirates is as high as 19 %. Unfortunately there are no enough data on the efficacy of this new class in Arab population, neither on the pattern of prescription. Our search for studies that evaluated the efficacy of these therapies in Arab population has revealed only one study. In this study the authors retrospectively assessed the effect of Sitagliptin in 53 UAE nationals and concluded that sitagliptin results in modest but important reductions in HbA1c and is associated with minimal side effects [9]. In this trial we aimed to evaluate the effect of Liraglutide in reducing HbA1c and weight on Arab population.

## Patients and methods

### Aim of the study

We aim to evaluate the efficacy of liraglutide as add on therapy to other anti-diabetic agents in reducing HbA1c and weight in Arab population with type2 diabetes.

### Primary end point

The primary end point of the study is to assess the change in weight and HbA1c from base line at 6 months.

### Secondary end point

We plan to assess the changes in lipid panel (total cholesterol and triglycerides), BP, creatinine from baseline and at 6 months.

### Study design

This prospective observational study was conducted at Dubai Hospital and two other centres in Dubai Health Authority, in the United Arab Emirates. The aim of this study was to assess the efficacy of liraglutide in reducing HbA1c and weight in Arabs with type2 diabetes. The study adhered to the tenets of the Declaration of Helsinki. The protocol was compliant with the Health Insurance Portability and Accountability Act and has been approved by the institutional review board and was given a reference number (MRC-08/2013_03). As per local recommendations, consent for participation in the study was obtained from all participants in the trial.

The primary end point of the study was the mean change in HbA1c and in weight from baseline and after 6 months of treatment. The changes in lipid panel from baseline to the end of follow-up were analysed as secondary end points, as were blood pressure (BP) and creatinine changes.

### Patient recruitment

All adult patients with type2 diabetes aged between 18 and 70 years, who were started on liraglutide in the period from January 2014 to March 2014 were recruited to the study. The prescription of liraglutide was compliant with the local recommendations; hence patients with type1 diabetes, severe renal impairment, pregnant women, and those with history of pancreatitis were not prescribed liraglutide. We also excluded non-Arab patients, and those who did not complete the period of six months on therapy. The dose of liraglutide started at 0.6 mg once per day subcutaneously and increased after one week to 1.2 mg and after another week to 1.8 mg per day. Those who did not tolerate the 1.2 mg were excluded from the study, while those who did not tolerate the 1.8 mg were advised to continue with 1.2 mg per day (1.2–1.8 mg were the doses used in LEAD trials).

After collecting informed consent, patients’ demographics (age, sex, and ethnic group), co-morbidities, concurrent medications, weight, blood pressure were recorded. Laboratory tests included fasting blood glucose, HbA1c, creatinine, lipid profile (Total cholesterol, Low-density lipoprotein, Triglycerides). Patients were then advised to attend for two visits in 3 months in which weight and HbA1c were collected. At 6 months (end of study visit) weight, blood pressure, HbA1c, creatinine, lipid profile and AST and ALT were recorded. Changes in pharmacologic therapy for hypertension and dyslipidaemia were allowed if it was deemed necessary and was left to the discretion of the treating physician.

#### Definitions

Patients were considered to have type2 diabetes if they fulfilled the ADA (American Diabetes Association) criteria for diagnosis of diabetes mellitus (FBG ≥126 mg/dl, RBG ≥200 mg/dl, or HbA1c ≥6.5 %) [10], or if they are already on anti-diabetic agents. Patients on metformin alone are not considered diabetic unless they fulfil the ADA (American Diabetes Association) criteria as many young ladies were on metformin for treatment of other medical problems e.g. polycystic ovarian syndrome. Hypertension was defined as a systolic blood pressure ≥140 mmHg and/or diastolic blood pressure ≥90 mmHg or being on antihypertensive medications.

### Data analysis

The population of our study included Arabs with type2 Diabetes mellitus who are obese or overweight. The sample size was calculated as 380 patients to provide a power of 95 % to detect the effects of liraglutide on weight and HbA1c.

Data analysis was performed on SPSS software 16.0. In all analysis, a *p*-value <0.05 was considered significant and *P* < 0.001 considered highly significant.

Quantitative variables were described as mean; SD and range, qualitative were described as variables as number and percentage. We have used the Chi-square test to compare qualitative variables between groups. The unpaired *t*-test was used to compare quantitative variables, in parametric data (SD < 50 % mean), while the Mann Whitney test was used instead of unpaired *t*-test in non-parametric data.

We used the Paired *t*-test to compare quantitative variable within the same group before and after in parametric data (SD < 50%mean), and the Willcoxon test was used instead of paired *t*-test in non-parametric data (SD > 50%mean).

## Results

Total number of patients screened was 463 patients; only 365 were recruited to the study and signed informed consent. The main reason for exclusion was patient’s unwillingness to sign an informed consent; furthermore, some patients were excluded, as they were lost to follow-up.

**Fig. 1:**
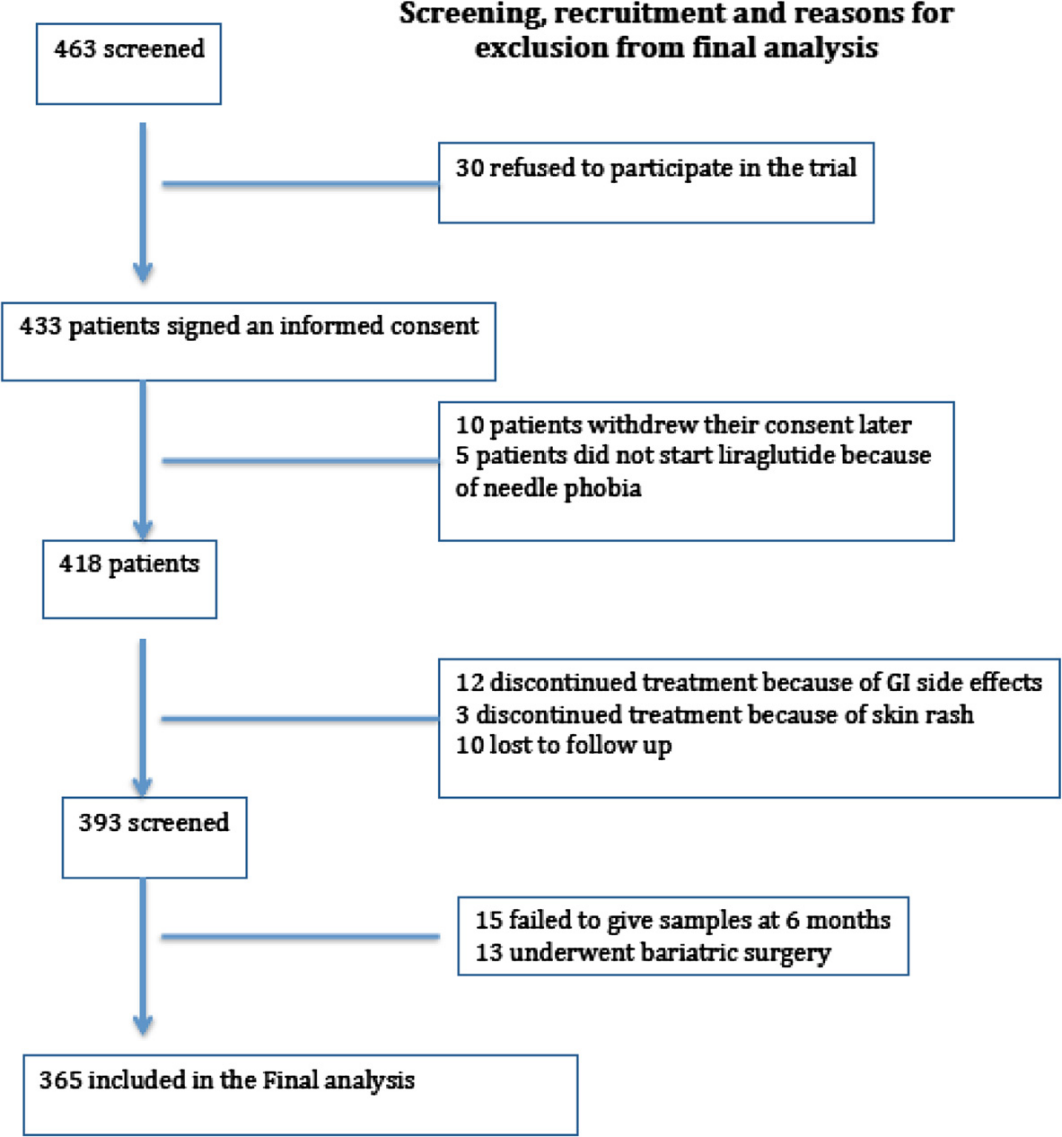
Screening, recruitment and reasons for exclusion from final analysis

Twelve patients stopped liraglutide in less than 2 months due to gastrointestinal side effects (3 patients did not tolerate increasing the dose of liraglutide from 1.2 to 1.8 mg and were allowed to continue the study); and another 3 developed skin rash following administration of liraglutide. The rash was macular in one patient and the other 2 developed wheals (urticaria) at the site of injection that disappeared within 48 h after stopping the injection. The rash recurred even after changing the needle and the pen. Five patients did not start the treatment or used only one or two doses after prescription because of needle phobia. Thirteen patients underwent bariatric surgery and had liraglutide discontinued post operatively. Fifteen patients failed to provide blood samples despite attending the clinics for re-fill prescription, 10 patients were lost to follow up, and finally 10 patients withdrew their consent (Fig. 1).

Out of the 365 patients included in the final analysis, 29 % were males (*n* = 106), and 71 % were females (*n* = 259). The mean age was 50.4 ± 10 years. Base line characteristics of participants are shown in (Table 1). At base line 56.34 % of patients were on insulin based regimen either basal-plus, basal bolus, or premixed. 90.1 % of patients were on metformin, 43.5 % were on sulphonyureas, 10.7 % were on DPP4 inhibitors, 0.8 % on Acarbose, and 85.2 % were on statins.

**Table 1:** Base line characteristics of participants

	Mean ± SD	Range
Males = 106 (29 %)		
Females = 259 (71 %)		
Age	50.4 ± 10	19–65
Weight	96.01 ± 19.2	60–165
HbA1c	8.3 ± 1.7	6–14
Total cholesterol	165.2 ± 40	77–335
Triglycerides	142.3 + 68	36–468
ALT	29 ± 18	4–117
AST	25.1 ± 20	7–315
Creatinine	0.77 ± 0.19	0.4–1.2
Systolic BP	131.6 ± 17	94–200
Diastolic BP	74.4 ± 10	43–110
Baseline medications	%	
Insulin based regimen	56.34	
Metformin	90.1	
Sulphonylureas	43.5	
DPP4 inhibitors	10.7	
Statins	85.2	
Antihypertensives	3.1	
Baseline comorbidities	%	
Dyslipidemia	26.6 %	
Hypertension	11 %	
Thyroid disorder	10 %	

**Fig. 2:**
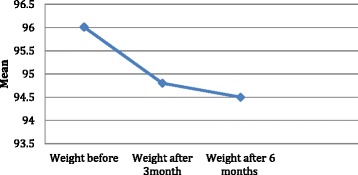
Changes in the body weight in kg among the studied cases

At base line the mean weight was 96.01 ± 19.2 kg; after 3 months of starting liraglutide the mean weight reduction was highly significant; 94.8 ± 20 kg with (*p* < 0.001)) and a percentage change of 2.01 ± 0.3 kg. At 6 months the mean weight was 94.5 ± 19 kg with (*p* < 0.001) and percentage change of 2.5 ± 0.6 kg; this was again highly significant. The change in weight was significant even when data was adjusted for age, gender, medications, and co-morbidities (Fig. 2).

**Fig. 3:**
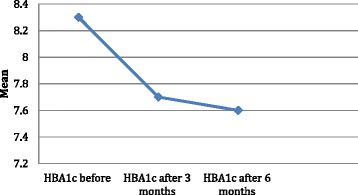
Changes in HBA1C in among the studied cases

Change in HbA1c was highly significant, with a change from baseline of 8.3 ± 1.7 to 7.7 ± 1.4 % (*p* < 0.001), and 7.6 + 1.6 % (*p* < 0.001) at 3 and 6 months respectively. Change in HbA1c was highly significant even after correction for age, gender, medications and co-morbidities (Fig. 3).

**Fig. 4:**
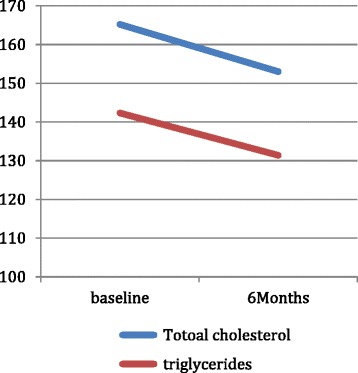
Changes in Total cholesterol and Triglycerides before and after therapy

There was a highly significant change in the total cholesterol level 6 months after starting treatment with liraglutide; the total cholesterol level has changed from 165.2 ± 40 mg/dl at baseline to 153.6 ± 35 mg/dl (*p* < 0.001) at 6 months. The same findings were seen with triglyceride, which has changed from 142.3 ± 68 mg/dl at baseline to 131.4 ± 61 mg/dl (*p* < 0.001) at 6 months (Fig. 4).

There was no significant change in systolic blood pressure at the end of the study. The diastolic blood pressure has dropped on therapy from a mean of 74.4 ± 10 mmHg at baseline to 72 ± 9 mmHg at 6 months, this was highly significant with *p* < 0.001. Serum creatinine has changes from 0.77 ± 0.19 mg/dl at base line to 0.76 ± 0.2 mg/dl at the end of the study. This change was not statistically significant. The *P* value was = 0.10.

## Discussion

Liraglutide is a GLP1 analogue that has proved very effective in reducing HbA1c not only as monotherapy as in LEAD 3 trial, but also in combination with oral hypoglycaemic agents as in LEAD 2 and LEAD 3. In all of these trials it resulted in 1.2–1.6 % reduction in HbA1c at doses of 1.2–1.8 mg. In LEAD 5 the percentage of patients achieving HbA1c <7 % and <6.5 % was higher when compared to those on metformin glimepiride and glargine [11].

In the Japanese population liraglutide was found to result in increased insulin secretion, suppression of glucagon, decreased glucose variability and a significantly reduced HbA1c, despite using a smaller dose (0.9 mg) [3]. Liraglutide when used as monotherapy compared to glimepiride in Japanese population resulting in superior HbA1c and weight reduction and was well tolerated [12]. Moreover it was found that the use of liraglutide resulted in a meaningful long-term weight loss and significantly improved eating behavior in obese Japanese patients with type2 diabetes [13]. In agreement with all previous trials, the use of liraglutide in our cohort (Arab population) has resulted in a significant reduction in HbA1c that ranged from 0.5 to 1.15 % in HbA1c in 6 months of therapy. These findings indicate that liraglutide is efficacious in lowering A1c and weight in Arab population and seems to have similar results to other trials in different races.

In their meta-analysis on the incretin based therapies in type2 Asian population, Melva et al. have concluded that out of all GLP-1 analogues exenatide was the only drug that resulted in significant weight loss in Asian population while liraglutide was weight neutral [14], these results were similar to data shown by Kendall et al. [15]. In the LEAD program the weight loss has ranged between 1.8 and 3.2 kg, depending on the dose of liraglutide and the combination therapy used in the program [11]. In our Cohort weight loss, it ranged between 0.5 and 17 kg with a mean weight loss of 2.5 ± 0.6 kg. Obesity can result in many adverse cardiovascular outcomes including heart failure and cardiomyopathy of obesity [16]. Weight reduction has a significant impact in the cardiovascular outcomes of patients with diabetes; this was proved in many studies that addressed the impact of surgically induced weight reduction on cardiovascular outcomes [17, 18], and more recently by Paul et al. who have shown that cardiovascular outcomes has significantly improved in patients receiving exenatide or exenatide plus insulin compared to those receiving insulin only [19].

Liraglutide therapy has resulted in significant reductions in total cholesterol and triglycerides in Arab population, total cholesterol has dropped from 165.2 ± 40 mg/dl at baseline to 153.6 ± 35 mg/dl (*p* < 0.001) at 6 months. Triglycerides have changed from 142.3 ± 68 mg/dl at baseline to 131.4 ± 61 mg/dl (*p* < 0.001) at 6 months. This finding is in keeping with previously published data that showed significant improvements in total cholesterol, and triglycerides compared with active comparators. The reduction in total cholesterol was 5.01 mg/dl from base line after liraglutide treatment, compared to a 1.93 mg/dl reduction for exenatide [20]. In a study in Japanese patients long-term use of liraglutide for 2 years found to maintain the reduction in body weight and glycemic control, and also improved lipid profile and liver enzymes [21].

Treatment with liraglutide was associated with significant reduction in systolic blood pressure from baseline achieved at 26 weeks were 2.59 mmHg (*p* = 0.008) and 2.49 mmHg (*p* = 0.003) [22]. In a meta-analysis by Wang B et al.; the use of liraglutide has resulted in significant reductions in systolic and diastolic blood pressures when compared to a placebo or glimepiride. In the 1.2 mg-treated group, liraglutide treatment reduced SBP compared with placebo and glimepiride treatment, with mean differences of −5.60 and −2.38 mmHg, and 95 % CIs of −5.84 to −5.36, *p* < 0.00001 and −4.75 to −0.01, *p* = 0.05, respectively. In the 1.8-mg-treated group, liraglutide also reduced SBP compared with placebo and glimepiride treatment with mean differences of −4.49 and −2.62 mmHg, and a 95 % CI of −4.73 to −4.26, *p* < 0.00001, and −2.91 to −2.33, *p* < 0.00001, respectively [23]. In our cohort there were no significant reductions in systolic blood pressure, but we noticed a significant reduction in diastolic blood pressure on therapy from a mean of 74.4 ± 10 to 72 ± 9 (mmHg) at 6 months this was highly significant with *p* < 0.001. This difference could not be explained with the available data. Further specific study might be required to confirm and explain, or disprove this finding.

Type2 diabetes often results in renal impairment that might interfere with the choice of medications used. Although liraglutide has a slightly wider evidence base than for exenatide or lixisenatide, there is no enough evidence to support its use in severe renal impairment [24]. Patients in our cohort were having normal renal function; those with stage III renal disease and beyond were excluded from the study. Liraglutide neither resulted in improvement or deterioration in kidney function.

### Limitations and strengths of the study

This is an observational study conducted to specifically assess the efficacy of liraglutide therapy in the Arab population in combination with other anti-diabetic agents. It is important to note that this is the only study thus far to report on the use of liraglutide in the Arab population and is a reflection of real-life clinical practice in this region. The sample size was calculated to give 95 % power to the study and was found to be 380. We have enrolled 463 but because of different reasons including side effects or loss to follow up, the final number was 363. Our study was not designed to look at safety and tolerability of liraglutide, which would have added a lot of value to the study.

## Conclusion

This is the first trial ever conducted to specifically look at the efficacy of liraglutide in Arab population; and in accordance with studies done in Caucasians, liraglutide therapy as an add-on to other anti-diabetic agents has proved very effective in reducing weight and HbA1c in Arab population with type2 diabetes; these beneficial effects were positive even after correction for age, sex, medications and co-morbidities. There was also a significant improvement in other metabolic parameters including total cholesterol and triglycerides as well as diastolic blood pressure and liver enzymes.
